# Dopaminergic Genetic Variation and Trait Impulsivity: The Role of *COMT* rs4680 in Mixed Behavioral and Substance Addictions

**DOI:** 10.3390/life15121836

**Published:** 2025-11-29

**Authors:** Gabriela Zdunek, Remigiusz Recław, Aleksandra Suchanecka, Krzysztof Chmielowiec, Dariusz Larysz, Marta Kuczak-Wójtowicz, Kinga Łosińska, Jolanta Chmielowiec, Anna Grzywacz

**Affiliations:** 1 Independent Laboratory of Behavioral Genetics and Epigenetics, Pomeranian Medical University in Szczecin, Powstancow Wielkopolskich 72 St., 70-111 Szczecin, Poland; zdugabriela@gmail.com (G.Z.); remigiusz.reclaw@pum.edu.pl (R.R.); aleksandra.suchanecka@pum.edu.pl (A.S.); martakuczak@wp.pl (M.K.-W.); 2 Department of Medical Sciences and Public Health, Gdansk University of Physical Education and Sport, Kazimierza Gorskiego 1 St., 80-336 Gdansk, Poland; kinga.losinska@awf.gda.pl; 3 Department of Hygiene and Epidemiology, Collegium Medicum, University of Zielona Góra, 28 Zyty St., 65-046 Zielona Góra, Poland; chmiele@vp.pl; 4 109th Military Hospital with Polyclinic, Ministry of National Defense, ul. Ksiedza Piotra Skargi 9/11, 71-422 Szczecin, Poland; dariuszlarysz@hotmail.com; 5 Department of Nursing, Collegium Medicum, University of Zielona Góra, 28 Zyty St., 65-046 Zielona Góra, Poland; j.chmielowiec@inz.uz.zgora.pl

**Keywords:** *COMT* rs4680, behavioral and substance addictions, impulsivity, BIS-11, gene-environment interaction, dopamine

## Abstract

**Background**: Impulsivity is a multidimensional trait associated with the development and maintenance of behavioral and substance addictions. Genetic polymorphisms, particularly within the dopaminergic system, are thought to modulate individual differences in impulsivity. The *COMT* rs4680 (Val158Met) polymorphism influences enzymatic activity of catechol-O-methyltransferase and may alter dopaminergic tone in the prefrontal cortex. This study investigated whether *COMT* rs4680 genotype interacts with addiction status (behavioral and substance addictions) to influence trait impulsivity. **Methods**: The study included 309 Polish men: 128 with mixed behavioral and substance addictions and 181 healthy controls. All participants completed the Barratt Impulsiveness Scale (BIS-11) and were genotyped for *COMT* rs4680. A two-way ANOVA was used to assess main and interaction effects of genotype and group on total and subscale BIS-11 scores. **Results**: Individuals with mixed addictions scored significantly higher on all BIS-11 subscales (*p* < 0.01). A significant interaction effect was observed for the Non-Planning (F_2,303_ = 4.40, *p* = 0.0131, η^2^ = 0.028) and Total BIS-11 scale (F_2,303_ = 5.77, *p* = 0.0035, η^2^ = 0.037), with the A/A genotype associated with increased impulsivity, especially among the clinical group. **Conclusions**: These findings support a gene-by-environment interaction in impulsivity, where *COMT* rs4680 Met/Met homozygotes may be more susceptible to heightened impulsivity in addiction contexts. The results highlight the potential utility of *COMT* genotyping in personalizing therapeutic strategies for impulse-related disorders such as addictive disorders. This study extends evidence on dopaminergic modulation of impulsivity to behavioral and substance addictions.

## 1. Introduction

Impulsivity is a multidimensional psychological construct defined by a tendency to act quickly and without adequate forethought, often leading to undesirable consequences. It is recognized as a stable personality trait with identifiable neurobiological and genetic correlates and plays a central role in the development and persistence of addictive disorders [1,2,3]. Among behavioral and substance addictions, gambling disorder has attracted particular attention, as it remains the only one formally recognized in major diagnostic systems such as the DSM-5 and ICD-11. Classified alongside substance use disorders due to overlapping clinical features and shared neurobiological mechanisms, gambling disorder provides a relevant model for studying impulsivity within reward-related dysfunctions [4,5,6].

A growing body of evidence points to the role of catecholaminergic pathways in the regulation of impulsivity [7,8]. One of the most studied genes in this context is the catechol-O-methyltransferase (*COMT*) gene, which encodes an enzyme responsible for degrading catecholamines, including dopamine, particularly in the prefrontal cortex. The common rs4680 (Val158Met) polymorphism results in a valine-to-methionine substitution at codon 158, leading to significantly lower enzymatic activity in Met/Met (A/A) homozygotes compared to Val/Val (G/G) individuals [9]. This polymorphism influences dopaminergic tone in brain regions involved in executive functioning, impulse control [10], and reward sensitivity.

Importantly, dopaminergic signaling in the prefrontal cortex follows an inverted U-shaped relationship with cognitive control: both insufficient and excessive dopamine levels can impair inhibitory functioning [11,12]. The *COMT* Val158Met polymorphism is therefore thought to modulate impulsivity by shifting individuals along this curve, with Val/Val carriers exhibiting relatively reduced dopamine tone and Met/Met carriers showing higher tonic dopamine activity [13,14]. While enhanced dopaminergic tone may improve working memory and cognitive stability in some contexts, it can also increase susceptibility to impulsive or risk-prone behaviors when reward cues are salient. Because dopaminergic signaling strongly influences reward sensitivity, decision-making, and inhibitory control, *COMT* variation is particularly relevant to impulsivity—a behavioral domain that mediates the transition from reward-driven behavior to compulsive addiction. In this sense, impulsivity may represent the most direct behavioral expression of prefrontal dopaminergic imbalance linked to *COMT* activity. This mechanistic framework provides a plausible neurobiological explanation for the inconsistent associations reported across studies of *COMT* and impulsivity [15].

Previous studies on *COMT* rs4680 and impulsivity have produced mixed results. While some report that the Met/Met genotype is linked to greater impulsivity, others find no significant effects or highlight the importance of contextual moderators such as clinical status or environmental exposure [16,17]. From a gene–environment interaction perspective, the influence of *COMT* on impulsivity may become more pronounced in risk-enhancing contexts, for example, when individuals engage in addictive behaviors.

Several studies have explored the association between *COMT* rs4680 and impulsivity across different populations, including healthy individuals, psychiatric patients, and those with substance use or behavioral addictions [17,18]. For instance, research in alcohol and stimulant dependence has linked the Met allele with higher impulsivity and reward sensitivity [17,18], while studies in pathological gambling and Internet gaming disorder have reported inconsistent or null effects. In non-clinical samples, *COMT* variation has been related to executive control, delay discounting, and decision-making under risk [9,10], but the direction of these associations appears to depend on environmental and contextual moderators [15]. These discrepancies indicate that *COMT* may not exert a uniform effect on impulsivity but rather interacts with individual or situational characteristics that shape its behavioral manifestation [15].

To reduce the influence of sex-related hormonal variability and neurobiological differences that are known to affect both *COMT* enzymatic activity and impulsivity traits, the study was restricted to male participants. Estrogen, for instance, has been shown to modulate *COMT* expression, which may confound analyses when both sexes are combined [19,20].

Beyond its contribution to understanding the neurogenetic mechanisms underlying impulsivity, this study has potential translational value. Identifying genotype-specific impulsivity profiles could enhance personalized approaches to treatment planning. For example, individuals with high-impulsivity genotypes may benefit from tailored cognitive-behavioral interventions focused on delay of gratification or from pharmacological strategies aimed at modulating dopaminergic function [21]. Moreover, interaction studies such as those linking *COMT* genotype and craving in relapse risk further support the relevance of genotype-guided therapeutic strategies [18].

Despite extensive research on *COMT* rs4680 and impulsivity [13,14,16,17], relatively few studies have examined this relationship in clinically defined mixed addiction populations, where both behavioral and substance-related mechanisms coexist. Most prior work has focused on isolated forms of addiction (e.g., gambling or alcohol use disorder) [5,6,18], limiting our understanding of how *COMT* variation operates across overlapping addictive behaviors. Furthermore, little is known about how specific impulsivity dimensions [1]—such as attentional, motor, and non-planning impulsivity—are differentially affected by *COMT* genotype in these high-risk groups. Addressing this gap may clarify the dopaminergic underpinnings of impulsivity across the behavioral–substance addiction spectrum [7,8,11,12].

The present study aimed to explore how trait impulsivity, as assessed with the Barratt Impulsiveness Scale (BIS-11), differs between men with mixed behavioral and substance addictions and non-addicted controls, and whether these differences are moderated by *COMT* rs4680 genotype. To the best of our knowledge, this is one of the first studies to examine the interaction between *COMT* rs4680 polymorphism, addiction status (behavioral and substance addictions), and multidimensional impulsivity in a male-only clinical sample, using a validated instrument such as the BIS-11. We hypothesized that men with mixed addictions would exhibit higher impulsivity than controls and that these differences would be moderated by the *COMT* rs4680 genotype.

## 2. Materials and Methods

### 2.1. Materials

The study included 309 male participants, comprising 128 individuals diagnosed with mixed behavioral and substance addictions (mean age = 27.73, SD = 5.74) and 181 healthy controls (mean age = 21.96, SD = 4.09). The analysis of the age difference between the study group and the control group and the lack of correlation with other analyzed characteristics are presented in Supplementary File S1. All participants were of Polish origin and were recruited from addiction treatment centers (clinical group, undergoing treatment for behavioral addiction) and through advertisements posted in university settings (control group). This recruitment strategy was chosen due to the accessibility of verified non-addicted individuals who had undergone standardized psychological screening within academic health programs. Although the university setting may introduce demographic differences such as age or education, these variables were statistically examined and controlled in subsequent analyses. Additional tests confirmed that these demographic factors did not significantly influence impulsivity scores. The clinical group was initially recruited for behavioral addiction (specifically gambling disorder). However, during the diagnostic interviews, it emerged that the vast majority of individuals in this group also met criteria for cannabis, tobacco, and amphetamine use disorders. Although this finding was unanticipated, it is fully consistent with the revised DSM-5 classification [4], which—unlike the previous DSM-IV—integrates gambling disorder with substance use disorders under the new DSM-5 category of Substance-Related and Addictive Disorders, commonly referred to as “addictive disorders”. This overlap reflects the increasingly recognized continuity between behavioral and substance addictions in terms of their shared dopaminergic and reward-related mechanisms. Consequently, the present study approached this clinical sample as representing a mixed addiction group, allowing for a more holistic examination of the neurobiological mechanisms underlying addiction rather than restricting the focus to a single diagnostic entity.

Exclusion criteria for both groups included intellectual disability, dementia, current psychoactive substance use disorder, abstinence from psychoactive substances or medications shorter than three months, neurodevelopmental disorders, history of traumatic brain injury, current suicide risk, and clinically significant somatic conditions (e.g., cardiovascular, endocrine, neurological, or metabolic disorders) that could affect cognitive functioning or mental health.

The protocol was approved by the Bioethics Committee of the Pomeranian Medical University in Szczecin (KB-0012/106/16). All participants provided written informed consent prior to inclusion in the study. The research was conducted at the Independent Health Promotion Laboratory.

Diagnosis of addictive disorders in the clinical group and confirmation of the absence of psychiatric disorders in the control group were carried out using the Mini International Neuropsychiatric Interview (MINI, version 5.0.0), a structured diagnostic interview consistent with DSM-IV criteria. Additionally, all participants completed the Barratt Impulsiveness Scale, Version 11 (BIS-11), which assesses various dimensions of impulsivity. The aim of the study was to assess potential interactions between behavioral traits and genetic variation in the catechol-O-methyltransferase gene (*COMT*, rs4680). Comparisons were made between individuals with mixed addictions and healthy controls to determine whether the Val158Met polymorphism (rs4680) modulated trait impulsivity.

### 2.2. Measures

Trait impulsivity was assessed using the Barratt Impulsiveness Scale, Version 11 (BIS-11), a widely used and validated self-report questionnaire designed to measure the personality and behavioral construct of impulsiveness. The BIS-11 consists of 30 items rated on a 4-point Likert scale ranging from 1 (“rarely/never”) to 4 (“almost always/always”). Higher scores indicate greater levels of impulsivity.

The BIS-11 provides a total score and three factor-derived subscales: Attentional Impulsivity (AI): difficulty focusing attention and cognitive instability. Motor Impulsivity (MI): acting without thinking or a tendency toward action without reflection. Non-Planning Impulsivity (NI): lack of forethought and consideration of future consequences. The questionnaire has been adapted and validated for the Polish population, with good psychometric properties. In the present study, the Barratt Impulsiveness Scale Version 11 (BIS-11) was administered under standardized conditions during individual assessments. The internal consistency of the scale was not recalculated in the current dataset.

To assess the internal consistency of the BIS-11 scale, Cronbach’s alpha coefficient was calculated. The scale demonstrated good reliability for all participants, with a Cronbach’s alpha value of 0.842, 95% CI [0.814, 0.870]. For the study group, Cronbach’s alpha was 0.848, 95% CI [0.801, 0.888] and for the control group, 0.832, 95% CI [0.789, 0.869]. This coefficient suggests that the scale is internally consistent and suitable for further analysis.

### 2.3. Genotyping

Genomic DNA was extracted from peripheral venous blood samples using standard salting-out procedures. Genotyping of the *COMT* rs4680 (Val158Met) polymorphism (dbSNP ID: rs4680) was performed using real-time polymerase chain reaction (PCR) with melting curve analysis. The genotyping protocol and assay parameters have been described in detail in a previous publication.

The amplification reactions were carried out using TIB MOLBIOL LightSNiP assays and a Roche LightCycler 480 system (Roche Diagnostics GmbH, Mannheim, Germany). Melting curves were generated by plotting fluorescence signal as a function of temperature to distinguish genotypes. Distinct melting peaks were observed at approximately 59.9 °C for the G (Val) allele and 53.3 °C for the A (Met) allele. Genotypes were automatically called using LightCycler 480 software version 1.5, and results were confirmed by visual inspection of the melting curve profiles [22].

All genotyping was performed at the Independent Laboratory of Health Promotion, and approximately 10% of randomly selected samples were re-genotyped to ensure accuracy. No discrepancies were found.

### 2.4. Statistical Analysis

Hardy–Weinberg equilibrium (HWE) for genotype distribution of the *COMT* rs4680 polymorphism was assessed using an online calculator (https://wpcalc.com/en/equilibrium-hardy-weinberg/, accessed on 19 June 2025). Group differences in genotype frequencies between individuals with behavioral addictions and healthy controls were evaluated using the chi-square test.

Differences in age between the study and control groups, as well as correlations with other analyzed variables, were examined using Pearson’s correlation and Principal Component Analysis (PCA).

The effects of *COMT* rs4680 genotype, group status (addicted vs. control), and their interaction on impulsivity traits were analyzed using factorial ANOVA models. Specifically, BIS-11 subscale and total scores were entered as dependent variables, with group and genotype as between-subjects factors. Interaction terms were included to assess potential gene-by-group effects.

Comparisons of the BIS scale between addicted and control participants were carried out using the Mann–Whitney U test for variables that did not meet normality assumptions.

Associations between the *COMT* rs4680 polymorphism and BIS-11 scores were further examined using multiple regression models. BIS-11 scales were entered as dependent variables, while age, behavioral addiction status, control group membership, *COMT* rs4680 genotype, and their interaction terms were included as predictors. A pseudo-coding method was applied for this polymorphism: GA heterozygotes served as the reference group, and homozygotes represented the effects on the dependent variables.

For variables related to the BIS-11 scale, a significance level of 0.0125 (0.05/4) was adopted using Bonferroni correction for multiple comparisons.

The assumption of homogeneity of variances was verified using Levene’s test (*p* > 0.05). Since some variables deviated from normal distribution, additional non-parametric comparisons were conducted using the Mann–Whitney U test. All statistical analyses were performed using STATISTICA version 13 (TIBCO Software Inc., Palo Alto, CA, USA) and for Windows (Microsoft Corporation, Redmond, WA, USA).

In the Supplementary Materials, correlation analyses were conducted using JASP version 0.95.2.0 (University of Amsterdam, The Netherlands), while all other statistical procedures were performed in STATISTICA.

## 3. Results

The genotype frequency distribution for the *COMT* rs4680 polymorphism was consistent with Hardy–Weinberg equilibrium (HWE) in both the mixed addiction group and the control group (Table 1).

No statistically significant differences were observed in the distribution of *COMT* rs4680 genotypes or allele frequencies between the mixed addiction group and the control group (Table 2).

Table 3 presents the means and standard deviations for all subscales and the total score of the Barratt Impulsiveness Scale Version 11 (BIS-11) in both the mixed addiction and control groups.

As shown in Table 3, participants in the mixed addiction group scored significantly higher than the control group on multiple dimensions of the BIS-11 scale, particularly Attentional Impulsivity and Motor Impulsivity, as well as on the Total Impulsivity score (all *p* < 0.001).

No statistically significant difference in impulsivity scores (BIS-11) was found between individuals with behavioral addictions and co-dependent on psychoactive substances and from education (Table 4).

The results of the 2 × 3 factorial ANOVA examining the interaction effects of *COMT* rs4680 genotype and group status (mixed addiction vs. control) on BIS-11 subscales and total scores are presented in Table 5. The influence of the following variables was additionally checked using multiple regression: mixed addiction vs. control, age, *COMT* rs4680 gene polymorphism [A/A] and [G/G] where heterozygotes [G/A] were the reference group (dummy variable), and the interactions of mixed addiction vs. control * age, mixed addiction vs. control * *COMT* rs4680 [A/A] and mixed addiction vs. control * *COMT* rs4680 [G/G] variables on the BIS-11 scale results.

BIS-AI scale

**Figure 1:**
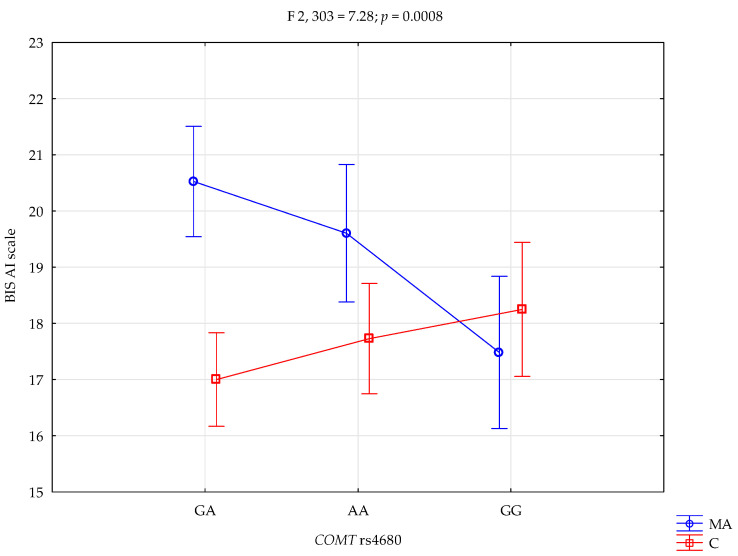
Interaction between *COMT* rs4680 genotype and group status (MA—mixed addiction vs. controls) on Attentional Impulsivity (BIS-11 AI).

A significant main effect of group status (mixed addiction vs. control) was observed on the BIS-Attentional Impulsivity (AI) subscale (F_1,303_ = 11.29, *p* < 0.0001, η^2^ = 0.036), with an observed power of 92%. This indicates that approximately 4% of the variance in BIS-AI scores can be attributed to group membership. Furthermore, a significant interaction effect was found between *COMT* rs4680 genotype and group status on BIS-AI scores (F_2,303_ = 7.28, *p* = 0.0008, η^2^ = 0.046; Figure 1), with an observed power of 94%, explaining around 5% of the score variance.

Post hoc comparisons (Table 6) revealed that individuals with mixed addictions carrying the A/A genotype scored significantly higher on the BIS-AI subscale compared to both their G/G counterparts within the addiction group and all genotype subgroups within the control group (A/A, G/A, and G/G). Similarly, those with the G/A genotype in the mixed addiction group also showed significantly elevated BIS-AI scores relative to addicted individuals with the G/G genotype and to controls with A/A and G/A genotypes.

A higher BIS-AI score was associated with mixed addiction status (β = 4.53; 95% CI [0.04, 9.02]; *p* = 0.04807) and the *COMT* rs4680 G/G genotype (β = 5.07; 95% CI [1.74, 8.40]; *p* = 0.00289). The interaction between the *COMT* rs4680 G/G genotype and mixed addiction status was associated with lower BIS-AI scores (β = −3.86; 95% CI [−6.06, −1.65]; *p* = 0.00066; Table 7).

BIS-MI scale

A significant main effect of group (mixed addiction vs. control) was found on the BIS-Motor Impulsivity (MI) subscale (F_1,303_ = 18.04, *p* < 0.0001, η^2^ = 0.056). The observed statistical power for this effect was 99%, with group status accounting for approximately 6% of the variance in BIS-MI scores.

A higher BIS-MI score was associated with the *COMT* rs4680 G/G genotype (β = 3.92; 95% CI [0.01, 7.83]; *p* = 0.04932). The interaction between the *COMT* rs4680 G/G genotype and mixed addiction status was associated with lower BIS-MI scores (β = −2.70; 95% CI [−5.30, −0.11]; *p* = 0.04133; Table 7).

BIS-NI scale

**Figure 2:**
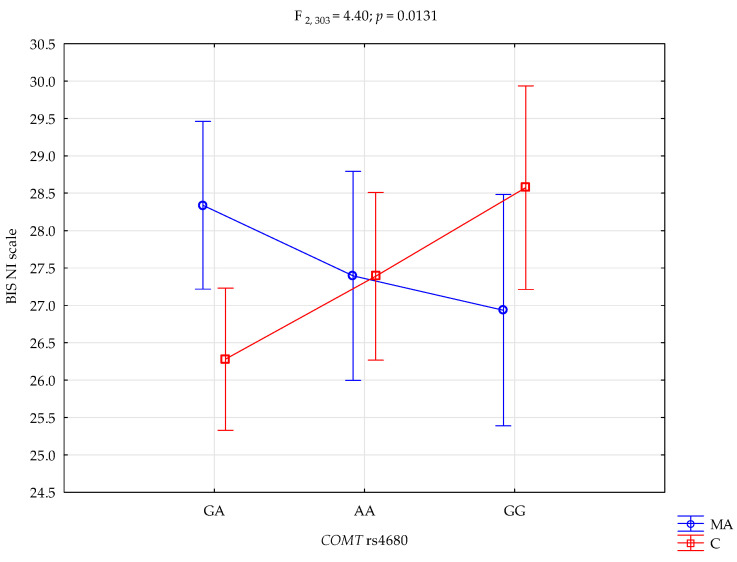
Interaction between *COMT* rs4680 genotype and group status (MA—mixed addiction vs. controls) on Non-Planning Impulsivity (BIS-11 NI).

A significant interaction effect was observed between the *COMT* rs4680 genotype and group status (behavioral addiction vs. control) on the BIS Non-Planning Impulsivity (NI) scores (F_2,303_ = 4.40, *p* = 0.0131, η^2^ = 0.028; Figure 2). The statistical power for this effect was 76%, indicating that approximately 3% of the variance in BIS-NI scores was attributable to the combined influence of genotype and group affiliation.

Post hoc analysis (Table 6) showed that participants with mixed addictions carrying the A/A genotype scored significantly higher on the BIS-NI scale (M = 28.34) than their A/A counterparts in the control group (M = 26.28; *p* = 0.0062). Additionally, control subjects with the G/G genotype exhibited significantly higher BIS-NI scores (M = 28.57) compared to A/A controls (M = 26.28; *p* = 0.0070). These results suggest that both the *COMT* rs4680 genotype and addiction status jointly modulate non-planning impulsivity, with increased vulnerability observed in A/A homozygotes among the mixed addiction group and in G/G homozygotes within the control group.

A higher BIS-NI scale was associated with the *COMT* rs4680 G/G genotype (β = 5.54; 95% CI [1.72, 9.36]; *p* = 0.00457). While the BIS-NI scale score decreases was associated with interaction the *COMT* rs4680 G/G genotype and the mixed addiction (β = −3.28; 95% CI [−5.82, −0.75]; *p* = 0.01134, Table 7).

BIS-11 Total scale

**Figure 3:**
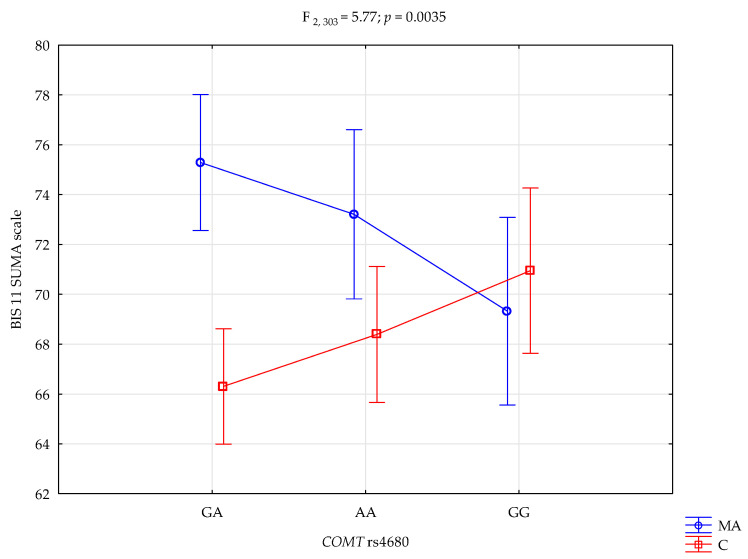
Interaction between *COMT* rs4680 genotype and group status (MA—mixed addiction vs. controls) on Total Impulsivity (BIS-11 Total Score).

A significant main effect of group status (mixed addictions vs. control) was observed for the BIS-11 total score (F_1,303_ = 10.09, *p* = 0.0016, η^2^ = 0.032), with an observed statistical power of 89%. Approximately 3% of the variance in impulsivity scores was attributable to group membership. Additionally, a significant interaction effect emerged between COMT rs4680 genotype and group status on the BIS-11 total score (F_2_,_303_ = 5.77, *p* = 0.0035, η^2^ = 0.037; Figure 3), with 87% statistical power and approximately 4% of the variance explained by this interaction.

Post hoc comparisons (Table 6) revealed that individuals with behavioral addictions carrying the A/A genotype scored significantly higher on the BIS-11 total scale than their G/G counterparts within the same group, as well as compared to all genotypic variants in the control group (A/A, A/G, and G/G). Furthermore, mixed addiction participants with the A/G genotype showed significantly higher total BIS-11 scores compared to controls with either the A/A or A/G genotype. Among the control group, participants with the G/G genotype had significantly higher total impulsivity scores than those with the A/A genotype.

A higher BIS-11 Total scale was associated with the *COMT* rs4680 G/G genotype (β = 14.29; 95% CI [4.99, 23.58]; *p* = 0.00269). While the BIS-11 Total scale score decreases was associated with interaction the *COMT* rs4680 G/G genotype and the mixed addiction (β = −9.74; 95% CI [−15.91, −3.57]; *p* = 0.00208, Table 7).

## 4. Discussion

The present study examined the interaction between *COMT* rs4680 polymorphism and mixed addiction status (here: gambling disorder) in shaping multidimensional impulsivity profiles. The most robust group effect was observed for the BIS-11 total score (F_1_,_303_ = 10.09, *p* = 0.0016, η^2^ = 0.032), while a significant genotype-by-group interaction emerged particularly for the Non-Planning Impulsivity subscale (F_2_,_303_ = 4.40, *p* = 0.0131, η^2^ = 0.028). As hypothesized, individuals with mixed addictions exhibited significantly higher impulsivity across BIS-11 subscales compared to non-addicted controls. Moreover, *COMT* genotype moderated these effects: the A/A variant (Met/Met) was consistently associated with elevated impulsivity scores, particularly within the clinical group. These findings support the notion that dopaminergic genetic variation may play a context-dependent role in the modulation of impulsive traits, reflecting differential expression across clinical and non-clinical populations rather than a classic gene-environment interaction.

From a neurobiological perspective, these findings are consistent with previous evidence highlighting the central role of prefrontal dopamine in regulating top-down control and response inhibition [23]. The *COMT* Val158Met (rs4680) polymorphism modulates enzymatic degradation of dopamine in the prefrontal cortex, with the Met/Met variant linked to reduced enzymatic activity and consequently higher dopaminergic tone [14,24]. Moderate increases in dopamine may enhance cognitive flexibility and motivation, whereas excessive levels—particularly in the context of dysfunctional reward circuitry—can impair inhibitory control and promote maladaptive decision-making. This inverted U-shaped dopamine–performance curve provides a plausible explanatory framework for the divergent behavioral outcomes observed across individuals [12,25]. However, the present study did not directly assess dopaminergic activity, prefrontal function, or cognitive performance. The reference to the inverted-U model is therefore conceptual, serving as a theoretical framework consistent with the prior literature rather than a mechanistic demonstration. Moreover, the absence of comparable effects in Val/Val homozygotes suggests that the relationship between *COMT* activity and impulsivity may not fully conform to a symmetrical inverted-U pattern.

Our results also support context-dependent models of genetic expression, which propose that genetic risk factors exert their strongest influence in adverse or activating environments [15,26]. In our study, the A/A genotype was associated with heightened impulsivity only among individuals with addiction, but not consistently in controls. This pattern suggests that the clinical and environmental context of addiction may unmask the behavioral expression of genetic vulnerability. Conversely, in non-clinical populations such as high-performance athletes, the same genotype may be linked to adaptive traits, including increased drive, novelty seeking, reward motivation, and rapid decision-making under pressure. In these contexts, impulsivity-like characteristics may serve functional roles, with dopaminergic variation supporting performance optimization rather than psychopathology. Such findings underscore the contextual plasticity of *COMT* rs4680, where identical genetic variants may manifest as pathological in addictive disorders but advantageous in high-functioning environments [27,28,29].

From a clinical perspective, our findings add to evidence implicating *COMT* rs4680 polymorphism in the neurobiological basis of addictive behaviors. Studies in substance use disorders have linked the Met/Met genotype to heightened reward sensitivity, impaired executive control, and reduced delay discounting—traits central to both substance-related and behavioral addictions [18,30,31,32]. Our results extend this framework to mixed behavioral and substance addictions, suggesting that dopaminergic modulation Via *COMT* may similarly influence impulsivity in non-substance addictions. Notably, this effect was most pronounced for attentional and non-planning impulsivity, dimensions closely tied to cognitive control and goal-directed behavior. Disruption of these facets in mixed addictions may help explain persistent engagement in risky activities despite negative consequences. This pattern aligns with broader evidence that impulsivity represents a transdiagnostic marker of addiction vulnerability, reflecting the interplay between dopaminergic regulation and behavioral control mechanisms.

Impulsivity has long been recognized as a core endophenotype of addictive disorders, linking dopaminergic dysregulation with maladaptive decision-making and relapse vulnerability. For example, in alcohol and stimulant dependence, impulsive behavior predicts both disorder onset and treatment outcomes [33]. Similarly, neuroimaging studies demonstrate that increased impulsivity is associated with altered activity in fronto-striatal networks involved in reward anticipation and cognitive control [34]. These findings further support the interpretation that COMT-related dopaminergic variation may influence addiction-related behavior primarily through its impact on prefrontal mechanisms of impulse regulation.

Our findings also provide empirical support for the DSM-5 classification, which groups behavioral addiction alongside substance use disorders based on shared neurobiological mechanisms. No group-level differences in *COMT* genotype or allele frequencies were observed between addicted and control participants. This suggests that *COMT* rs4680 does not confer a direct genetic risk for developing addiction, but rather modulates behavioral expression within individuals already affected. Such modulatory effects are consistent with the polygenic and environmentally contingent nature of addictive behaviors, where individual variants exert subtle, context-dependent influences. While these findings may have theoretical relevance for understanding individual variability in treatment response, their potential clinical application remains highly preliminary and requires prospective pharmacogenetic validation.

Although preliminary, these findings raise questions relevant to future research on personalized treatment strategies. Individuals with mixed addictions carrying the Met/Met (A/A) genotype, who showed greater impulsivity, might benefit from interventions aimed at strengthening cognitive control and modulating dopaminergic tone. Potential approaches include cognitive remediation, mindfulness-based relapse prevention, or pharmacological agents targeting dopamine pathways. Aripiprazole, a partial dopamine D2 receptor agonist, has been studied as a treatment for addictive disorders [35] and may be especially useful in individuals with altered prefrontal dopaminergic regulation. Likewise, bupropion, a norepinephrine–dopamine reuptake inhibitor, has shown promise in improving executive control and reducing craving in populations with dopaminergic dysfunction, particularly in nicotine dependence [36,37]. However, its potential role in mixed addictions remains largely unexplored. Evidence remains preliminary, genotype-specific treatment studies are scarce, and further research is needed to evaluate the efficacy and safety of such personalized approaches. Other pharmacological agents such as opioid antagonists (e.g., naltrexone) have also shown efficacy in addictive disorders and may represent alternative targets for genotype-informed treatment research [38].

Beyond clinical settings, the same dopaminergic mechanisms discussed here may also be relevant to motivational and reward-driven behaviors observed in sport and achievement contexts. Although not directly examined in the present study, this conceptual parallel reflects a broader continuum of dopaminergic functioning—from maladaptive impulsivity in addiction to adaptive motivation and persistence in competitive environments. Such speculation aligns with the “differential susceptibility” framework, which emphasizes that the same genetic variants may confer vulnerability or advantage depending on environmental context [28,29,39].

This dual interpretation reflects the concept of differential susceptibility, in which genetic variants act not as fixed risk factors but as plasticity factors that heighten sensitivity to both adverse and supportive environments. In this framework, the *COMT* rs4680 polymorphism may represent a neurogenetic marker of behavioral adaptability, shaped by the dynamic interplay of biology, personality, and environmental context.

### Translational Implications and Limitations

The observed genotype-related differences in impulsivity may have theoretical relevance for developing personalized prevention and treatment approaches in addictive disorders. Individuals carrying the Met/Met (*COMT* rs4680 A/A) genotype—who in our study showed higher levels of non-planning and attentional impulsivity—may require targeted interventions to strengthen cognitive control and reduce impulsive tendencies. Although speculative, future studies could examine whether pre-treatment genotyping might inform the choice of therapeutic strategies such as cognitive-behavioral interventions focused on inhibitory control, or pharmacological agents targeting dopaminergic regulation (e.g., aripiprazole). Such approaches remain hypothetical and would require rigorous pharmacogenetic validation before any clinical application.

More broadly, our findings illustrate how neurobiological variability interacts with environmental exposures to shape psychological traits. The observed genotype-by-group interaction underscores the importance of context-specific effects, whereby the same genetic profile may confer risk in one domain (e.g., addictive disorders) but advantages in another (e.g., sport). This perspective supports the ongoing shift in psychiatric genetics from risk-based to plasticity-based models.

Nonetheless, several limitations should be noted. First, the exclusive inclusion of male participants, although methodologically justified and necessary to control for sex-related hormonal influences on *COMT* expression, limits the generalizability of our findings. Because estrogen modulates *COMT* activity, future research should examine potential sex-specific effects in female samples. Another limitation concerns sample representativeness. The comparison group was recruited from university settings, whereas the clinical group came from addiction treatment centers. This introduces potential demographic and environmental differences (e.g., age, education, socioeconomic background) that may influence impulsivity independently of genotype. However, age was statistically controlled in all regression models, and additional analyses showed no significant effects of age or education on BIS-11 scores. Although these controls reduce the likelihood of bias, future studies should recruit demographically matched groups to minimize residual confounding. Second, while the BIS-11 is a widely used self-report tool, it does not capture all dimensions of impulsivity, particularly those best assessed with behavioral tasks or neuroimaging. Third, although our sample size was sufficient to detect moderate interaction effects, replication in larger and more diverse populations is needed to strengthen robustness and external validity. Finally, although the interaction effects between *COMT* genotype and addiction status reached statistical significance, the corresponding effect sizes were modest. These small effects likely reflect the multifactorial nature of impulsivity, which is shaped by numerous genetic, environmental, and psychological influences. Therefore, the present findings should be interpreted with caution and validated in larger, multivariable models integrating both biological and environmental factors. The cross-sectional design precludes causal inferences about the direction of observed associations.

Another limitation concerns the diagnostic profile of the clinical group. Although participants were initially recruited for gambling disorder, structured interviews revealed concurrent substance use disorders (cannabis, tobacco, and amphetamine) in all cases. While this comorbidity limits the ability to attribute effects exclusively to gambling-related pathology, it also reflects the real-world overlap between behavioral and substance addictions recognized in DSM-5. Consequently, this mixed addiction sample offers a more integrated perspective on dopaminergic mechanisms that cut across different forms of addiction, providing valuable insight into the shared biological substrate of impulsivity and addictive behavior. Nonetheless, this diagnostic heterogeneity may have introduced uncontrolled variance and should be considered a major limitation when interpreting genotype–phenotype relationships. The modest effect sizes observed further underscore that *COMT* rs4680 exerts only subtle modulatory influences, with limited immediate clinical applicability.

Overall, the present findings should be regarded as preliminary, hypothesis-generating evidence that can inform—but not yet guide—clinical translation. Future studies should integrate genotyping with multimodal assessments, including functional neuroimaging and behavioral tasks, to clarify the neurobiological pathways linking *COMT* variation, impulsivity, and addictive behavior. Longitudinal designs may also help determine whether *COMT* genotype influences the course of addictive disorders and treatment response over time.

## 5. Conclusions

This study shows that trait impulsivity in mixed behavioral and substance addictions is moderated by the *COMT* rs4680 polymorphism, with the Met/Met (A/A) genotype associated with heightened non-planning and attentional impulsivity. These findings support the hypothesis that genetic variation in dopaminergic modulation contributes to the individual differences in impulse control among patients with addictive disorders. The observed gene-by-environment interaction emphasizes the relevance of personalized approaches in the assessment and treatment of addictive behaviors. Genotyping of *COMT* rs4680 may, in the future, support the development of individualized therapeutic strategies—both behavioral and pharmacological—tailored to neurogenetic profiles.

## Figures and Tables

**Table 1:** Hardy–Weinberg equilibrium for *COMT* rs4680 genotype distribution in individuals with mixed addictions and healthy controls.

Genotypes		Observed (Expected)	Allele Freq	χ^2^ (*p*-Value)
*COMT* rs4680				
Mixed addiction n = 128	A/A	38 (35.6)	*p* (A) = 0.53 q (G) = 0.47	0.727 (0.3939)
	G/A	59 (63.8)		
	G/G	31 (28.6)		
control n = 181	A/A	59 (55.2)	*p* (A) = 0.55 q (G) = 0.45	1.272 (0.2594)
	G/A	82 (89.5)		
	G/G	40 (36.2)		

*p*—statistical significance χ^2^ test.

**Table 2:** Frequency distribution of *COMT* rs4680 genotypes and alleles in the mixed addiction group and healthy controls.

*COMT* rs4680					
	Genotypes			Alleles	
	A/A n (%)	G/A n (%)	G/G n (%)	A n (%)	G n (%)
Mixed addiction n = 128	38 (29.69%)	59 (46.09%)	31 (24.22%)	135 (52.73%)	121 (47.26%)
Control n = 181	59 (32.60%)	82 (45.30%)	40 (22.10%)	200 (55.25%)	162 (44.75%)
χ^2^ (*p* value)	0.3590 0.8357			0.3819 (0.5366)	

n—number of subjects.

**Table 3:** Differences in impulsivity scores (BIS-11) between individuals with mixed addictions and healthy controls.

BIS-11 Scale	Mixed Addictions	Control	Z	(*p*-Value)
BIS-AI	19.52 ± 4.34	17.51 ± 3.57	4.386	0.0001 #
BIS-MI	26.00 ± 4.83	23.45 ± 4.15	4.806	0.0001 #
BIS-NI	27.72 ± 4.83	27.15 ± 4.11	1.159	0.2465
BIS-11 Total	73.23 ± 12.00	68.01 ± 9.82	4.160	0.0001 #

*p*—statistical significance (Mann–Whitney U test); #—Bonferroni correction applied (α = 0.05/4 = 0.0125).

**Table 4:** Differences in BIS-11 impulsivity scores between individuals with behavioral and substance-related addictions and according to education level.

Comorbid Substance Dependence n (%)	BIS-AI Scale M; Yes vs. No [Z; *p*-Value]	BIS-MI Scale M; Yes vs. No [Z; *p*-Value]	BIS-NI Scale M; Yes vs. No [Z; *p*-Value]	BIS-11 Total Scale M; Yes vs. No [Z; *p*-Value]
Opiates 27 (21%)	20.15 vs. 19.35 [1.297; *p* = 0.1947]	26.74 vs. 25.80 [0.821; *p* = 0.4119]	27.78 vs. 27.70 [0.245; *p* = 0.8062]	74.63 vs. 72.85 [0.902; *p* = 0.3669]
Cannabinole 93 (73%)	19.68 vs. 19.09 [−0.706; *p* = 0.4804]	26.40 vs. 24.94 [−1.622; *p* = 0.1047]	27.86 vs. 27.34 [−0.834; *p* = 0.4043]	73.94 vs. 71.34 [−1.406; *p* = 0.1597]
Cocaine 12 (9%)	20.50 vs. 19.41 [−0.789; *p* = 0.4302]	27.25 vs. 25.87 [−0.965; *p* = 0.3347]	27.08 vs. 27.78 [0.298; *p* = 0.7654]	74.83 vs. 73.06 [−0.768; *p* = 0.4422]
Sedatives and sleeping pills 15 (12%)	20.33 vs. 19.41 [1.148; *p* = 0.2509]	26.20 vs. 25.97 [0.067; *p* = 0.9468]	27.13 vs. 27.80 [−0.115; *p* = 0.9086]	73.67 vs. 73.17 [0.293; *p* = 0.7698]
Stimulants 96 (75%)	19.14 vs. 20.66 [−1.731; *p* = 0.0835]	25.85 vs. 26.44 [−0.572; *p* = 0.5671]	27.47 vs. 28.47 [−1.197; *p* = 0.2314]	72.46 vs. 75.53 [−1.310; *p* = 0.1903]
Hallucinogenic 13 (10%)	19.23 vs. 19.55 [0.406; *p* = 0.6846]	26.85 vs. 25.90 [−0.560; *p* = 0.5754]	29.15 vs. 27.56 [−1.183; *p*= 0.2367]	75.23 vs. 73.00 [−0.513; *p* = 0.6081]
primary schools 64 (50%) vs. secondary 59 (46%) and higher 5 (4%) education	20.09 vs. 18.94 [−1.315; *p*= 0.1884]	26.08 vs. 25.92 [−0.396; *p* = 0.6924]	28.02 vs. 27.42 [−0.577; *p* = 0.5642]	74.19 vs. 72.27 [−0.715; *p* = 0.4747]

*p*—statistical significance (Z—Mann–Whitney U test); M—mean.

**Table 5:** Results of factorial ANOVA for *COMT* rs4680 genotype, group status, and their interaction on BIS-11 subscales and total score.

BIS-11	Group	*COMT* rs4680				ANOVA		
		A/A n = 97 M ± SD	A/G n = 141 M ± SD	G/G n = 71 M ± SD	Factor	F (*p*-Value)	η^2^	Power (alfa = 0.05)
BIS-AI	Mixed addictions (MA); n = 128	19.61 ± 3.72	20.53 ± 4.04	17.48 ± 4.97	intercept MA/control *COMT* rs4680 MA/control × *COMT*	F_1,303_ = 6424.47 (*p* < 0.0001) *# F_1,303_ = 11.29 (*p* < 0.0001) *# F_2,303_ = 1.36 (*p* = 0.2588) F_2,303_ = 7.28 (*p* = 0.0008) *#	0.955 0.036 0.009 0.046	1.000 0.918 0.292 0.935
	Control; n = 181	17.73 ± 3.41	17.00 ± 3.50	18.25 ± 3.86				
BIS-MI	Mixed addictions (MA); n = 128	26.21 ± 5.00	26.42 ± 4.59	24.94 ± 5.07	intercept MA/control *COMT* rs4680 MA/control × *COMT*	F_1,303_ = 8612.50 (*p* < 0.0001) *# F_1,303_ = 18.04 (*p* < 0.0001) *# F_2,303_ = 0.02 (*p* = 0.9822) F_2,303_ = 2.32 (*p* = 0.1001)	0.966 0.056 0.0001 0.015	1.000 0.988 0.053 0.469
	Control; n = 181	23.27 ± 4.42	23.13 ± 3.86	24.37 ± 4.27				
BIS-NI	Mixed addictions (MA); n = 128	27.39 ± 4.80	28.34 ± 4.42	26.94 ± 5.56	intercept MA/control *COMT* rs4680 MA/control × *COMT*	F_1,303_ = 10941.17 (*p* < 0.0001) *# F_1,303_ = 0.07 (*p* = 0.7882) F_2,303_ = 0.25 (*p* = 0.7811) F_2,303_ = 4.40 (*p* = 0.0131) *	0.973 0.0002 0.001 0.028	1.000 0.058 0.089 0.756
	Control; n = 181	27.39 ± 4.51	26.28 ± 3.79	28.57 ± 3.74				
BIS-11 Total	Mixed addictions (MA); n = 128	73.21 ± 11.34	75.29 ± 10.76	69.32 ± 14.25	intercept MA/control *COMT* rs4680 MA/control × *COMT*	F_1,303_ = 12202.32 (*p* < 0.0001) *# F_1,303_ = 10.09 (*p* = 0.0016) *# F_2,303_ = 0.10 (*p* = 0.9017) F_2,303_ = 5.77 (*p* = 0.0035) *#	0.976 0.032 0.0007 0.037	1.000 0.886 0.066 0.867
	Control; n = 181	68.39 ± 10.84	66.30 ± 8.94	70.95 ± 9.46				

MA—Mixed addictions; *—significant result; M ± SD—mean ± standard deviation; n—number of subjects; *p*—statistical significance (ANOVA test); η^2^—effect size (partial eta squared). #—Bonferroni correction applied (α = 0.05/4 = 0.0125).

**Table 6:** Post hoc comparisons (Least Significant Difference) for BIS-AI, BIS-NI, and BIS-11 total scores across *COMT* rs4680 genotypes and group status.

***COMT* rs4680 and BIS-AI**						
	{1} M = 20.52	{2} M = 19.60	{3} M = 17.48	{4} M = 17.00	{5} M = 17.73	{6} M = 18.25
Mixed addiction A/A {1}		0.2492	0.0004 *	0.0001 *	0.0001 *	0.0040 *
Mixed addiction A/G {2}			0.0229 *	0.0006 *	0.0192 *	0.1195
Mixed addiction G/G {3}				0.5497	0.7734	0.4040
Control A/A {4}					0.2661	0.0918
Control A/G {5}						0.5071
Control G/G {6}						
***COMT* rs4680 and BIS-NI**						
	{1} M = 28.34	{2} M = 27.39	{3} M = 26.93	{4} M = 26.28	{5} M = 27.39	{6} M = 28.57
Mixed addiction A/A {1}		0.3006	0.1495	0.0062 *	0.2400	0.7926
Mixed addiction A/G {2}			0.6650	0.1957	0.9957	0.2350
Mixed addiction G/G {3}				0.4785	0.6403	0.1187
Control A/A {4}					0.1388	0.0070 *
Control A/G {5}						0.1873
Control G/G {6}						
***COMT* rs4680 and BIS-11 Total**						
	{1} M = 75.29	{2} M = 73.21	{3} M = 69.32	{4} M = 66.30	{5} M = 68.39	{6} M = 70.95
Mixed addiction A/A {1}		0.3489	0.0120 *	0.0001 *	0.0005 *	0.0476 *
Mixed addiction A/G {2}			0.1323	0.0011 *	0.0303 *	0.3493
Mixed addiction G/G {3}				0.179810	0.6931	0.5234
Control A/A {4}					0.2522	0.0244 *
Control A/G {5}						0.2413
Control G/G {6}						

*—significant statistical differences; M—mean. {1} Mixed addiction A/A; {2} Mixed addictions A/G; {3} Mixed addictions G/G; {4} Control A/A; {5} Control A/G; {6} Control G/G.

**Table 7:** Multiple regression analysis of BIS-11 scores with *COMT* rs4680 genotype (G/A as reference), age, and group status as predictors, including their interaction terms.

β (*p*)	BIS-AI Scale	BIS-MI Scale	BIS-NI Scale	BIS-11 Total Scale
**reference** β [−95% CI, +95% CI] *p* value	15.52 [8.55, 22.48] *p* = 0.00002 *#	23.73 [15.54, 31.93] *p* < 0.00001 *#	26.21 [18.20, 34.21] *p* < 0.00001 *#	65.12 [45.64, 84.59] *p* < 0.00001 *#
**MA/C** β [−95% CI, +95% CI] *p* value	4.53 [0.04, 9.02] *p* = 0.04807 *	1.36 [−3.93, 6.64] *p* = 0.61399	3.01 [−2.15, 8.17] *p* = 0.25158	9.06 [−3.50, 21.62] *p* = 0.15689
**Age** β [−95% CI, +95% CI] *p* value	−0.13 [−0.42, 0.16] *p* = 0.39128	−0.18 [−0.53, 0.17] *p* = 0.3127	−0.13 [−0.47, 0.21] *p* = 0.46919	−0.43 [−1.26, 0.40] *p* = 0.31022
***COMT* rs4680 [A/A]** β [−95% CI, +95% CI] *p* value	2.49 [−0.50, 5.48] *p* = 0.10177	0.66 [−2.86, 4.17] *p* = 0.71380	3.27 [−0.16, 6.70] *p* = 0.06138	6.63 [−1.72, 14.98] *p* = 0.11903
***COMT* rs4680 [G/G]** β [−95% CI, +95% CI] *p* value	5.07 [1.74, 8.40] *p* = 0.00289 *#	3.92 [0.01, 7.83 ] *p* = 0.04932 *	5.54 [1.72, 9.36] *p* = 0.00457 *#	14.29 [4.99, 23.58] *p* = 0.00269 *#
MA/C * Age β [−95% CI, +95% CI] *p* value	−0.01 [−0.19, 0.17] *p* = 0.90513	0.09 [−0.12, 0.30] *p* = 0.40640	−0.01 [−0.22, 0.20] *p* = 0.92503	0.07 [−0.44, 0.57] *p* = 0.79599
MA/C * *COMT* rs4680 [A/A] β [−95% CI, +95% CI] *p* value	−1.63 [−3.64, 0.38] *p* = 0.11121	−0.43 [−2.79, 1.93] *p* = 0.71809	−2.04 [−4.34, 0.27] *p* = 0.08337	−4.21 [−9.82, 1.41] *p* = 0.14134
MA/C * *COMT* rs4680 [G/G] β [−95% CI, +95% CI] *p* value	−3.86 [−6.06, −1.65] *p* = 0.00066 *#	−2.70 [−5.30, −0.11] *p* = 0.04133 *	−3.28 [−5.82, −0.75] *p* = 0.01134 *#	−9.74 [−15.91, −3.57] *p* = 0.00208 *#

MA—Mixed addiction group; C—control group; reference (reference category in the regression model); β (regression coefficient); CI (confidence interval, −95% CI; +95% CI); *p* (statistical significance level); * (statistically significant difference, *p* < 0.05); #—Bonferroni correction applied (α = 0.05/4 = 0.0125).

## Data Availability

The data presented in this study are available on request from the corresponding author. The data are not publicly available due to privacy concerns.

## Supplementary Materials

The following supporting information can be downloaded at https://www.mdpi.com/article/10.3390/life15121836/s1. Table S1. Principal Component Analysis (PCA) of BIS-11 impulsivity scores across COMT rs4680 genotypes in mixed addiction and control groups, with age included as a covariate; Table S2. Pearson’s correlations between impulsivity scores (BIS-11) and age; Figure S1. Principal Component Analysis (PCA) of BIS-11 impulsivity scores, COMT rs4680 genotype, age, and group status (mixed addictions vs. controls); Figure S2. Heatmap of Pearson’s correlations between impulsivity scores (BIS-11) and age; Figure S3. Pearson’s correlations between impulsivity scores (BIS-11) and age; Figure S4. Scatter plot of age across COMT rs4680 genotypes (GA, AA, GG) in the mixed addiction (MA) and control (C) groups; Figure S5. Scatter plot of BIS-AI scores across COMT rs4680 genotypes (GA, AA, GG) in the mixed addiction (MA) and control (C) groups; Figure S6. Scatter plot of BIS-MI scores across COMT rs4680 genotypes (GA, AA, GG) in the mixed addiction (MA) and control (C) groups; Figure S7. Scatter plot of BIS-NI scores across COMT rs4680 genotypes (GA, AA, GG) in the mixed addiction (MA) and control (C) groups; Figure S8. Scatter plot of BIS-11 total scores across COMT rs4680 genotypes (GA, AA, GG) in the mixed addiction (MA) and control (C) groups.
