# Supplementary material for: Dopaminergic Genetic Variation and Trait Impulsivity: The Role of COMT rs4680 in Mixed Behavioral and Substance Addictions
Source: Life (Basel). 2025 Nov 29;15(12):1836. doi: 10.3390/life15121836 (PMC12733793; doi:10.3390/life15121836)
Supplement: Supplementary file 1 [file life-15-01836-s001.zip › life-3902922-supplementary.pdf]

Suplement 1.

Table S1. Principal Component Analysis (PCA) of BIS-11 impulsivity scores across *COMT* rs4680 genotypes in mixed addiction and control groups, with age included as a covariate.

Variable	Factor 1	Factor 2	Factor 3
**MA / C**	0.339	-0.795	0.135
**Age**	-0.049	0.883	0.010
***COMT* rs4680**	-0.012	0.131	0.983
**BIS-AI**	-0.858	-0.080	-0.091
**BIS-MI**	-0.853	0.064	-0.015
**BIS-NI**	-0.803	-0.252	0.139
**BIS-11 Total**	-0.993	-0.099	0.012
**Eigenvalue**	3.211	1.513	1.013
**Variance Explained**	0.459	0.216	0.145

MA - Mixed addictions group; C - control group

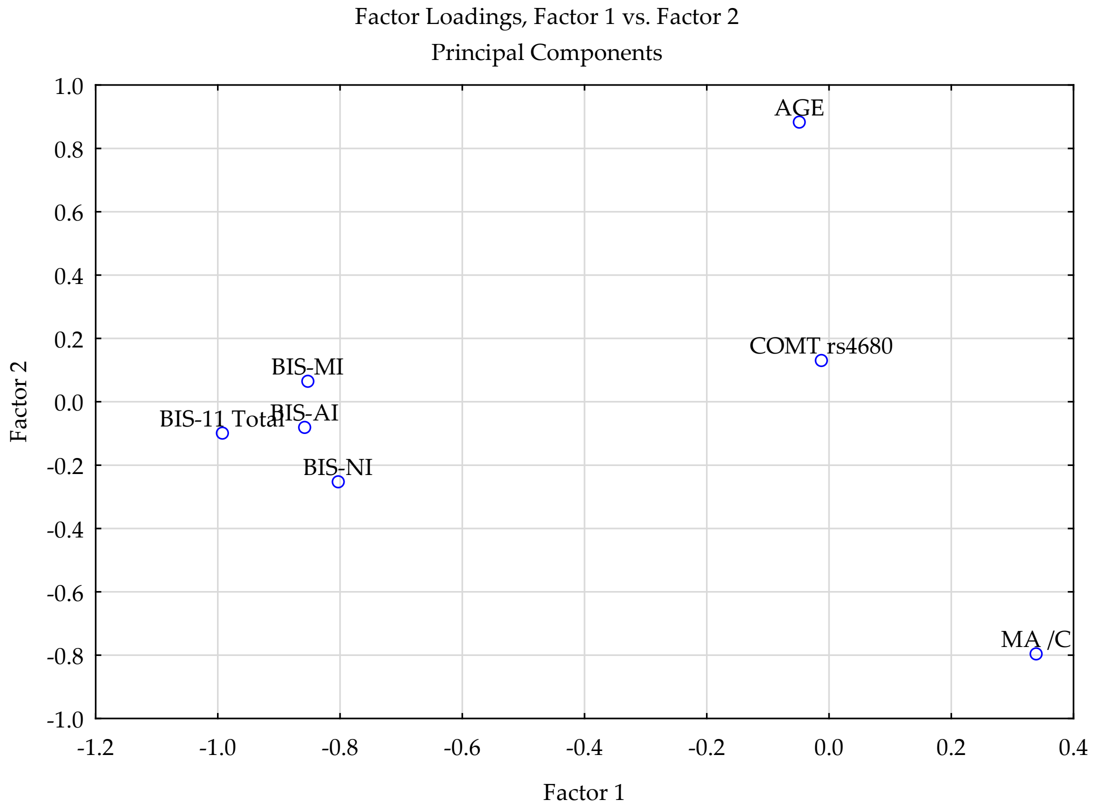

Figure S1. Principal Component Analysis (PCA) of BIS-11 impulsivity scores, *COMT* rs4680 genotype, age, and group status (mixed addictions vs. controls). Factor 1 reflects impulsivity dimensions, while Factor 2 captures age-related variation.

Table S2. Pearson's correlations between impulsivity scores (BIS-11) and age.

			r	*p*	
BIS-AI	-	BIS-MI	0.603	***	< .001
BIS-AI	-	BIS-NI	0.573	***	< .001
BIS-AI	-	BIS-11 Total	0.849	***	< .001
BIS-AI	-	Age	-0.046		.419
BIS-MI	-	BIS-NI	0.529	***	< .001
BIS-MI	-	BIS-11 Total	0.853	***	< .001
BIS-MI	-	Age	0.092		.107
BIS-NI	-	BIS-11 Total	0.823	***	< .001
BIS-NI	-	Age	-0.107		.059
BIS-11 Total	-	Age	-0.018		.750

Significance levels: * $p < .05$, ** $p < .01$, *** $p < .001$

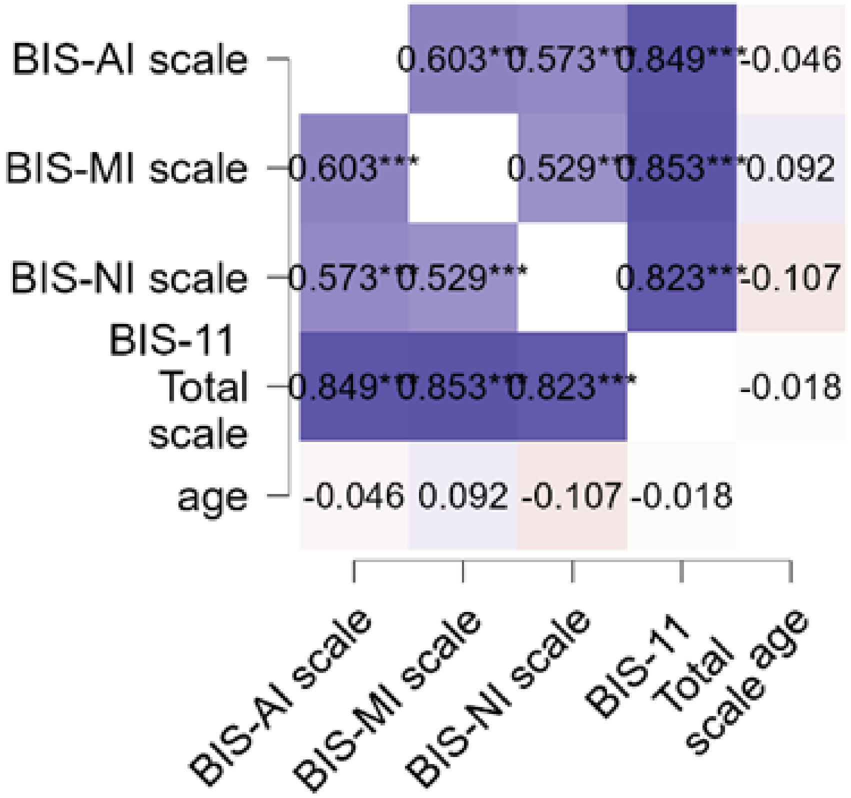

Figure S2. Heatmap of Pearson's correlations between impulsivity scores (BIS-11) and age.

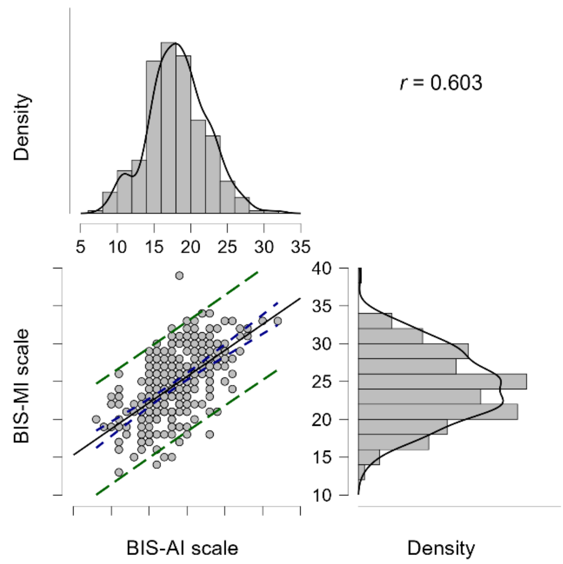

r = 0.603
Density
BIS-MI scale
BIS-AI scale
Density

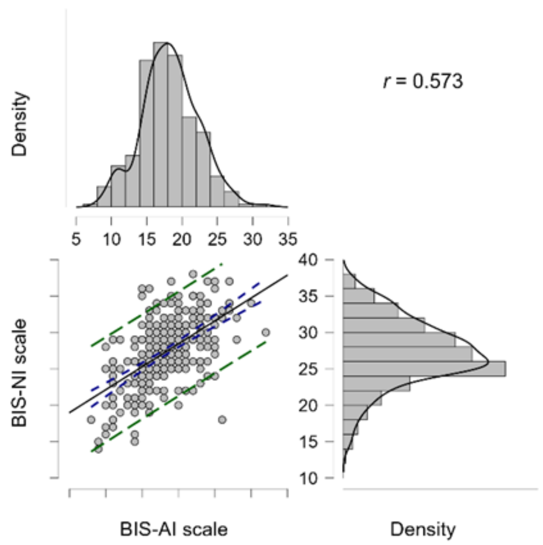

r = 0.573
Density
BIS-NI scale
BIS-AI scale
Density

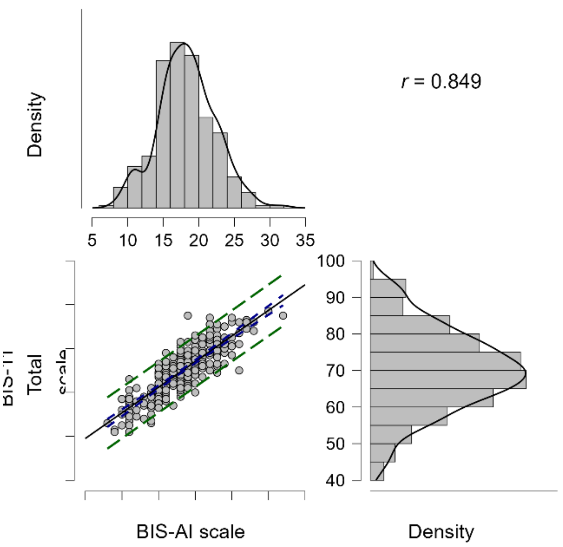

r = 0.849
Density
Total
BIS-AI scale
Density

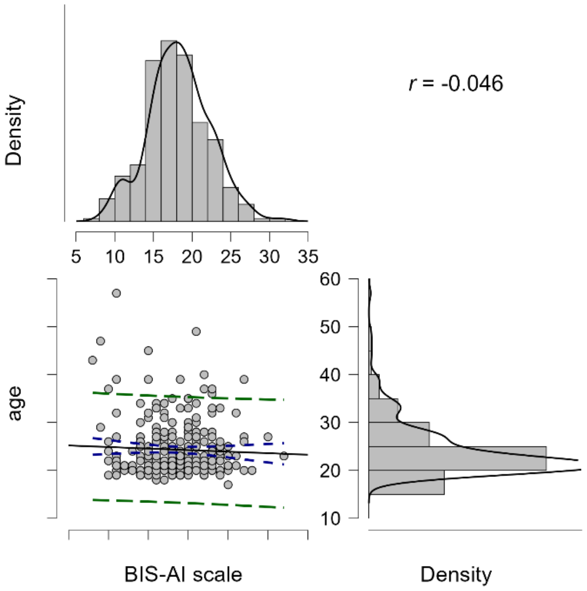

r = -0.046
Density
age
BIS-AI scale
Density

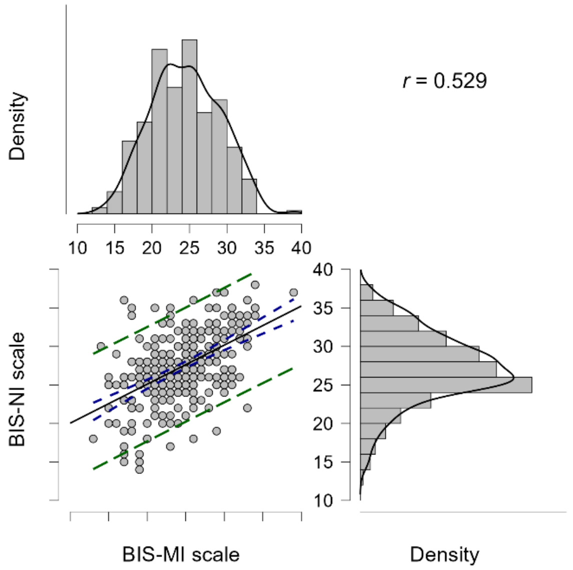

r = 0.529
Density
BIS-NI scale
BIS-MI scale
Density

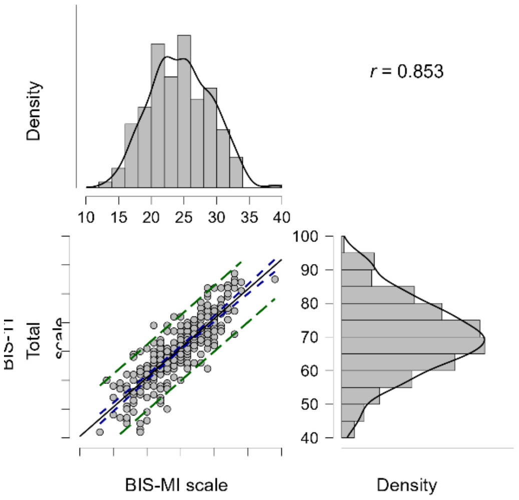

r = 0.853
Density
Total
BIS-MI scale
Density

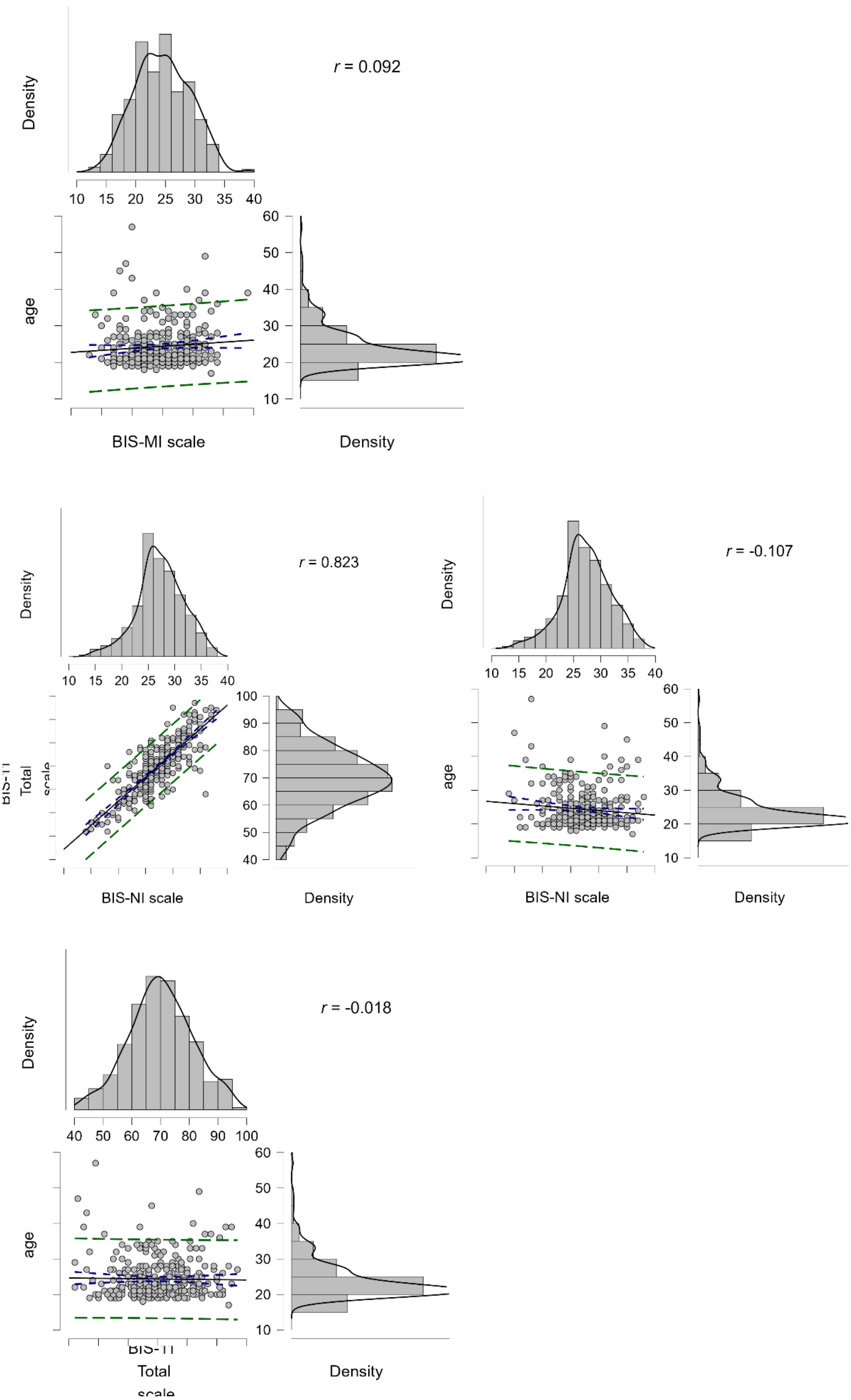

Figure S3. Pearson's correlations between impulsivity scores (BIS-11) and age.

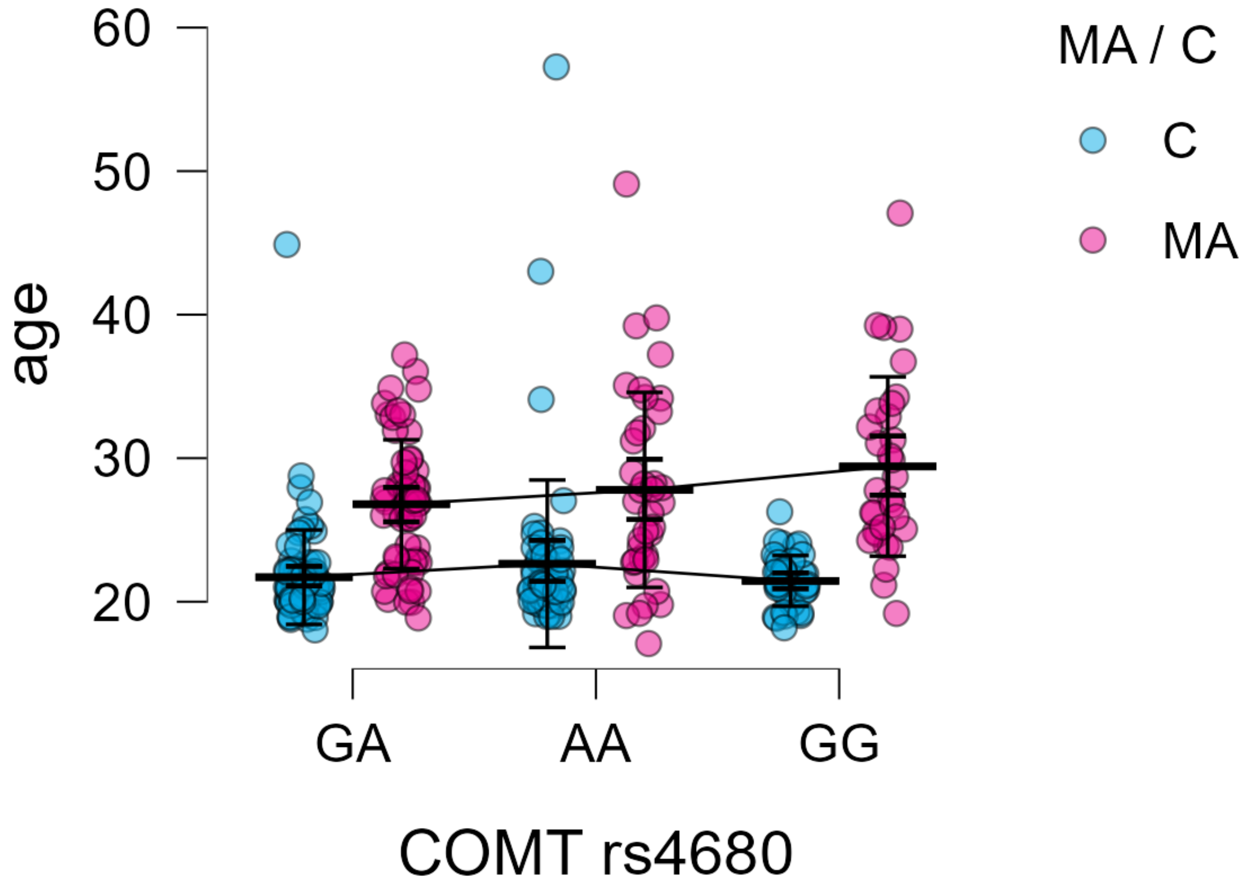

Figure S4. Scatter plot of age across *COMT* rs4680 genotypes (GA, AA, GG) in the mixed addiction (MA) and control (C) groups. Each point represents an individual participant, with mean and standard error bars shown.

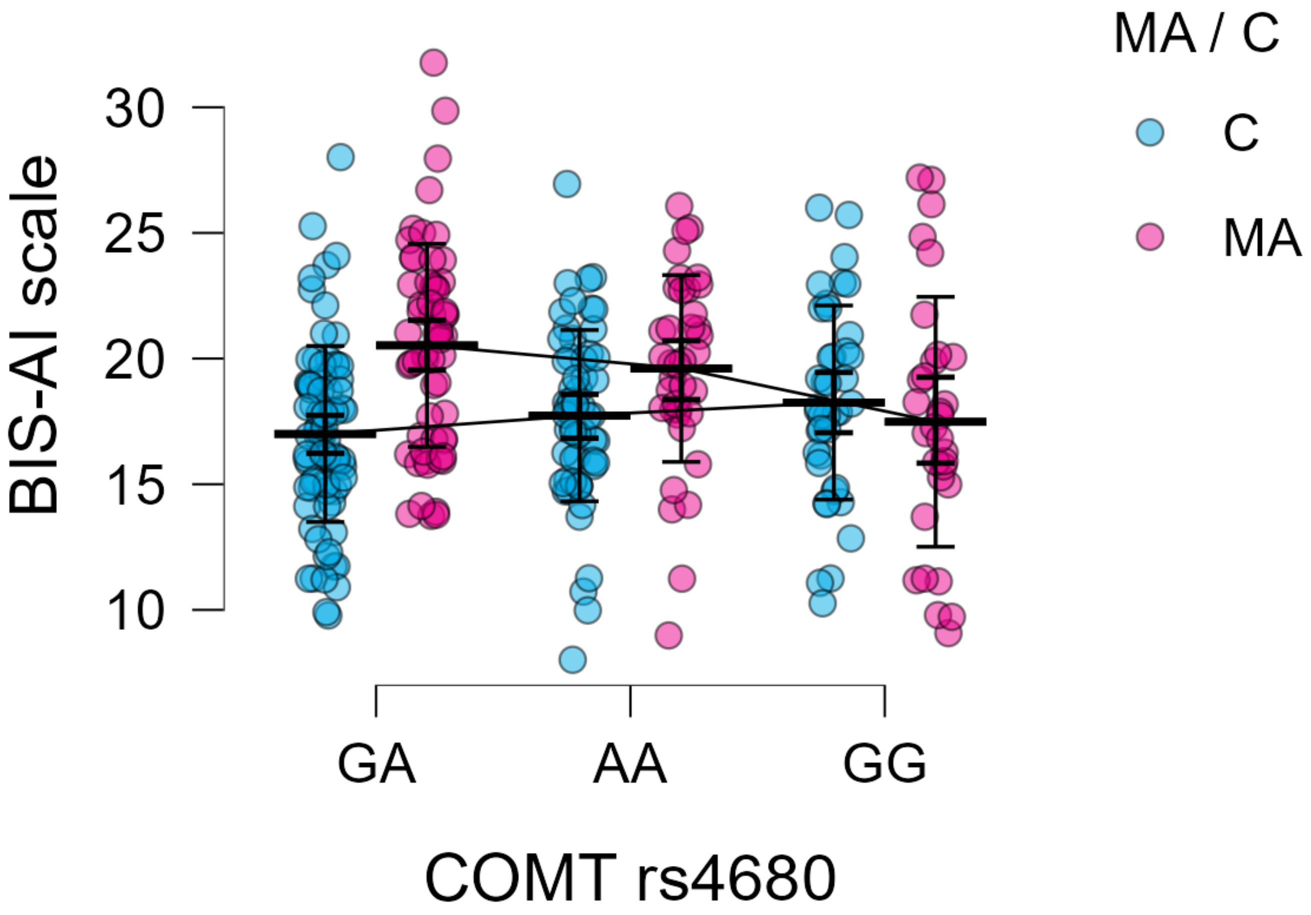

Figure S5. Scatter plot of BIS-AI scores across *COMT* rs4680 genotypes (GA, AA, GG) in the mixed addiction (MA) and control (C) groups. Each point represents an individual participant, with mean and standard error bars shown.

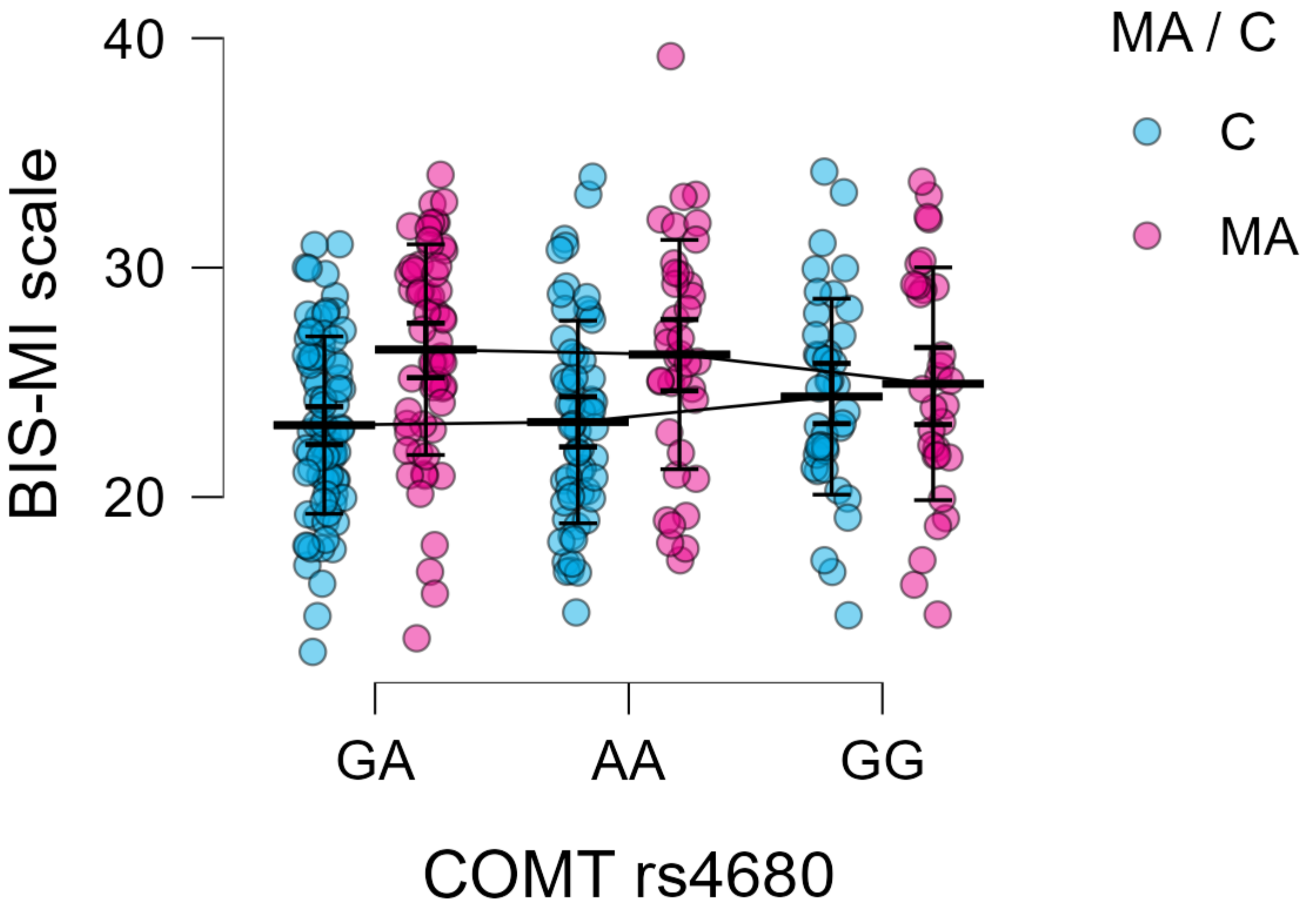

Figure S6. Scatter plot of BIS-MI scores across *COMT* rs4680 genotypes (GA, AA, GG) in the mixed addiction (MA) and control (C) groups. Each point represents an individual participant, with mean and standard error bars shown.

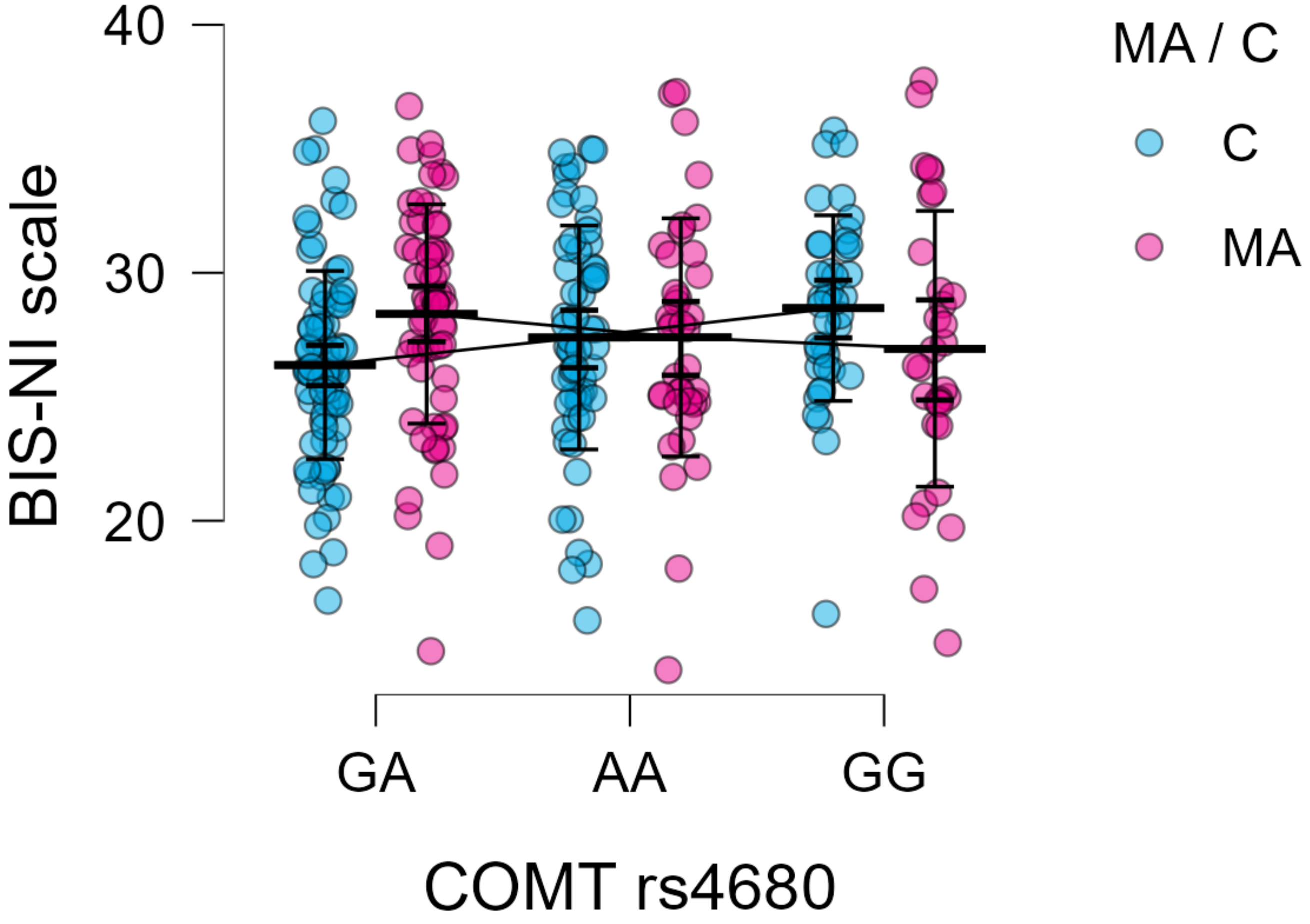

Figure S7. Scatter plot of BIS-NI scores across *COMT* rs4680 genotypes (GA, AA, GG) in the mixed addiction (MA) and control (C) groups. Each point represents an individual participant, with mean and standard error bars shown.

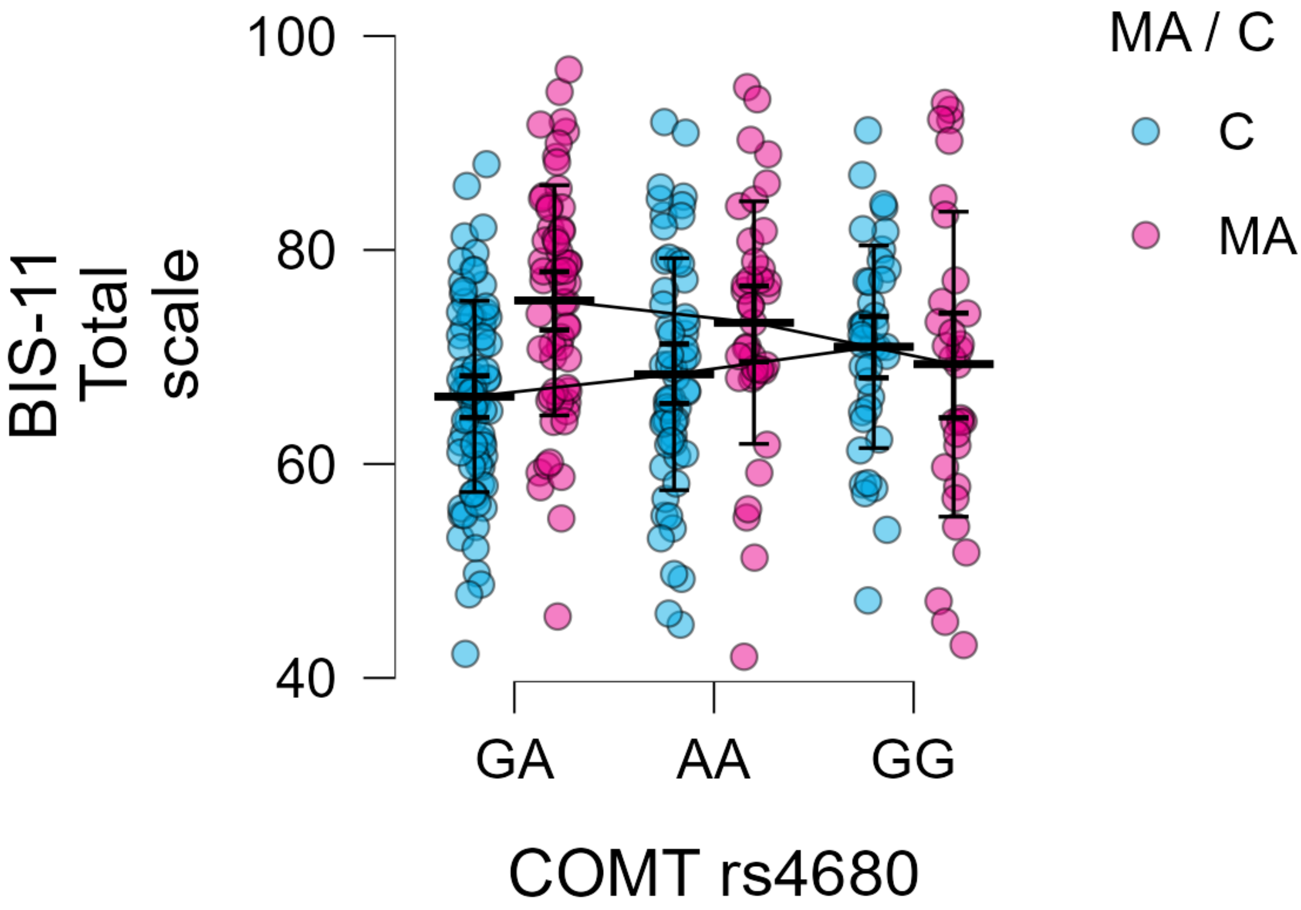

Figure S8. Scatter plot of BIS-11 total scores across *COMT* rs4680 genotypes (GA, AA, GG) in the mixed addiction (MA) and control (C) groups. Each point represents an individual participant, with mean and standard error bars shown.
